# Astaxanthin Prevents Diet-Induced NASH Progression by Shaping Intrahepatic Immunity

**DOI:** 10.3390/ijms222011037

**Published:** 2021-10-13

**Authors:** Ming Yang, Eric T. Kimchi, Kevin F. Staveley-O’Carroll, Guangfu Li

**Affiliations:** 1 Department of Surgery, University of Missouri, Columbia, MO 65212, USA; yangmin@health.missouri.edu (M.Y.); kimchie@health.missouri.edu (E.T.K.); 2 Harry S. Truman Memorial VA Hospital, Columbia, MO 65201, USA; 3 Department of Molecular Microbiology and Immunology, University of Missouri, Columbia, MO 65212, USA

**Keywords:** non-alcoholic fatty liver disease, non-alcoholic steatohepatitis, astaxanthin, intrahepatic immune response, treatment, nanoparticles

## Abstract

Dietary change leads to a precipitous increase in non-alcoholic fatty liver disease (NAFLD) from simple steatosis to the advanced form of non-alcoholic steatohepatitis (NASH), affecting approximately 25% of the global population. Although significant efforts greatly advance progress in clarifying the pathogenesis of NAFLD and identifying therapeutic targets, no therapeutic agent has been approved. Astaxanthin (ASTN), a natural antioxidant product, exerts an anti-inflammation and anti-fibrotic effect in mice induced with carbon tetrachloride (CCl_4_) and bile duct ligation (BDL); thus, we proposed to further investigate the potential effect of ASTN on a diet-induced mouse NASH and liver fibrosis, as well as the underlying cellular and molecular mechanisms. By treating pre-development of NASH in mice induced with a choline-deficient, L-amino acid-defined, high-fat diet (CDAHFD), we have demonstrated that oral administration ASTN preventively ameliorated NASH development and liver fibrosis by modulating the hepatic immune response, liver inflammation, and oxidative stress. Specifically, ASTN treatment led to the reduction in liver infiltration of monocyte-derived macrophages, hepatic stellate cell (HSC) activation, oxidative stress response, and hepatocyte death, accompanied by the decreased hepatic gene expression of proinflammatory cytokines such as TNF-α, TGF-β1, and IL-1β. In vitro studies also demonstrated that ASTN significantly inhibited the expression of proinflammatory cytokines and chemokine CCL2 in macrophages in response to lipopolysaccharide (LPS) stimulation. Overall, in vivo and in vitro studies suggest that ASTN functions as a promising therapeutic agent to suppress NASH and liver fibrosis via modulating intrahepatic immunity.

## 1. Introduction

Non-alcoholic fatty liver disease (NAFLD) is a broad spectrum of liver disease, affecting approximately 25% of the global population [1]. Due to a growing impact on world health, there has been explosive interest in NAFLD. The histological manifestations of NAFLD range from simple liver steatosis to aggressive non-alcoholic steatohepatitis (NASH) characterized by ballooning hepatocyte injury, lobular and/or portal inflammation associated with or without liver fibrosis [2]. NAFLD-associated metabolic comorbidities include obesity, type 2 diabetes (T2D), hypertension, and hyperlipidemia, and metabolic syndrome [3]. NAFLD and its comorbidities are often associated with severe diseases, such as chronic kidney disease [4] and hospitalized cardiovascular disease [5]. The prevalence of NAFLD is increasing similar to the incidence of obesity. Young adults and children with NAFLD have higher rates to develop long-term mortal diseases, including cancers and cardiometabolic diseases [6]. Although many anti-obesity and anti-diabetic drugs are under investigation, no agent is approved yet for NAFLD treatment [7]. Without preventive therapy and appropriate treatment, NAFLD or NASH may progress to liver cirrhosis and hepatocellular carcinoma (HCC) [3,8]. 

**Figure 1:**
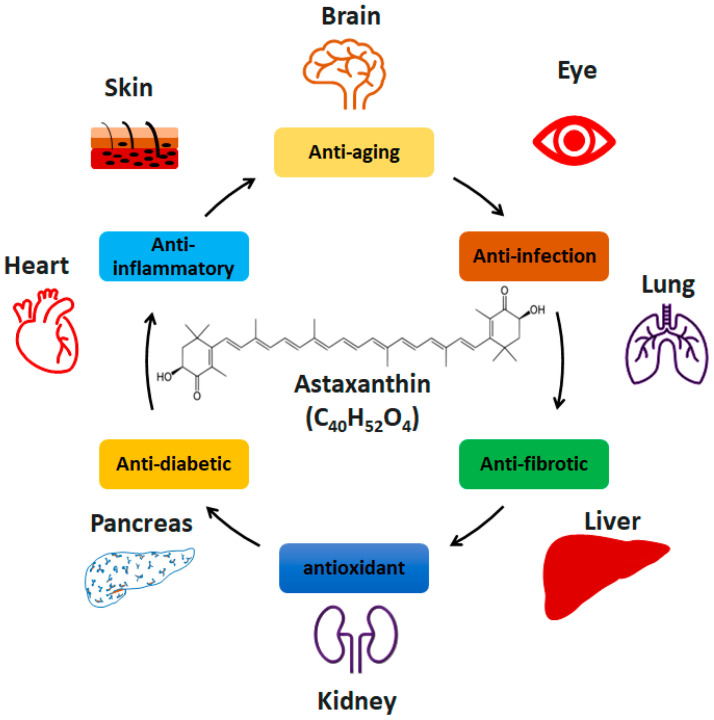
The function of astaxanthin in different diseases.

Inappropriate inflammatory response and oxidative stress have been identified as two major pathological factors causing NAFLD/NASH. Natural anti-inflammatory and antioxidant products have been broadly investigated in the treatment of NAFLD/NASH [9]. Astaxanthin (ASTN, 3,3′-dihydroxy-β, β′-carotene-4,4′-dione) is a red carotenoid pigment produced naturally in green microalgae *Haematococcus pluvialis* [10] and *Chlorella zofingiensis* [11], and heterobasidiomycetous yeast *Xanthophyllomyces dendrorhous* [12]. It shows a broad spectrum of properties, including anti-inflammatory, anti-diabetic, antioxidant, anti-aging, and anti-cancer activities [13,14,15]. The application of ASTN has been investigated in different diseases from various organs and tissues, including skin [16], brain [17], eye [18], heart [19], lung [20], kidney [21], and pancreas [22] (Figure 1). The potential functions of ASTN have been investigated in clinical trials (https://clinicaltrials.gov (accessed on 1 September 2021)), such as oxidative stress in patients with polycystic ovary syndrome (NCT03991286) and skin aging (NCT03460860). The results of clinical trials aiming to evaluate the bioactivity of ASTN are expected.

Recent findings show that ASTN exerts a preventive effect on liver inflammation and fibrosis. For example, Shen et al. also reported that ASTN can prevent liver fibrosis by inhibiting the activation of hepatic stellate cells (HSCs) via decreasing transforming growth factor (TGF)-β1 expression and by downregulating autophagy in fibrotic livers from mice treated with carbon tetrachloride (CCl_4_) or bile duct ligation (BDL) [23]. These functions of ASTN were associated with potential roles of anti-apoptosis of liver cells and anti-inflammation function in macrophages, evidenced by in vitro studies on liver cell line NCTC1469 cells and mouse macrophage cell line RAW264.7 cells. The in vivo effects of ASTN in immune response remain to be detected. In addition, ASTN treatment can improve diet-induced systemic metabolic disorders in mice by reducing blood glucose levels and serum levels of total triglyceride and cholesterol [24]. However, how ASTN modulates hepatic immune response in mice with NASH and liver fibrosis and the underlying cellular and molecular mechanisms are not fully understood. 

Numerous dietary models have been applied to mimic human NAFLD/NASH progression. Currently, there is no single rodent model that can reflect all the spectrum of human disease progression, but each model can imitate some important features of human NASH [25]. In this study, we chose a choline-deficient, L-amino acid-defined, high-fat diet (CDAHFD, A06071302, Research Diets Inc.) to induce mouse NASH model, which can quickly and consistently develop liver fibrosis, steatosis, and inflammation [26], some typical features of human NASH. In addition, feeding this diet can promote NASH-HCC progression in mice [27]. CDAHFD diet consists of 60 kcal% fat and 0.1% methionine by weight [26], abbreviated to HFD in the following context of this study. Eight-week-old C57BL/6 mice were fed this HFD for 6 weeks to induce mouse NASH with rapid liver fibrosis development. The intrahepatic immune profile was analyzed in the mice fed with normal diet (ND) or HFD with or without oral treatment of ASTN. Feeding HFD induced liver inflammatory and fibrotic NASH, which accompanied the infiltration of inflammatory cells, including CD8^+^ T cells, macrophages, and myeloid cells. ASTN treatment significantly inhibited the infiltration of monocyte-derived macrophages and prevented liver inflammation and injury, resulting in retarded development of NASH and liver fibrosis. Molecular studies demonstrated that ASTN as antioxidant and anti-inflammatory reagents also can inhibit profibrotic and pro-inflammatory gene expression, oxidative stress response, and the expression of fibroblast growth factor (FGF) and extracellular matrix (ECM) genes in the NASH liver. These findings suggest that the infiltration of monocytes-derived macrophages mediates the progression of NASH and ASTN treatment can modulate the intrahepatic immune response to inhibit NASH development.

## 2. Results

### 2.1. Characterization of NASH Pathogenesis in Mice Induced by a Choline-Deficient, L-Amino Acid-Defined, High-Fat Diet (CDAHFD)

**Figure 2:**
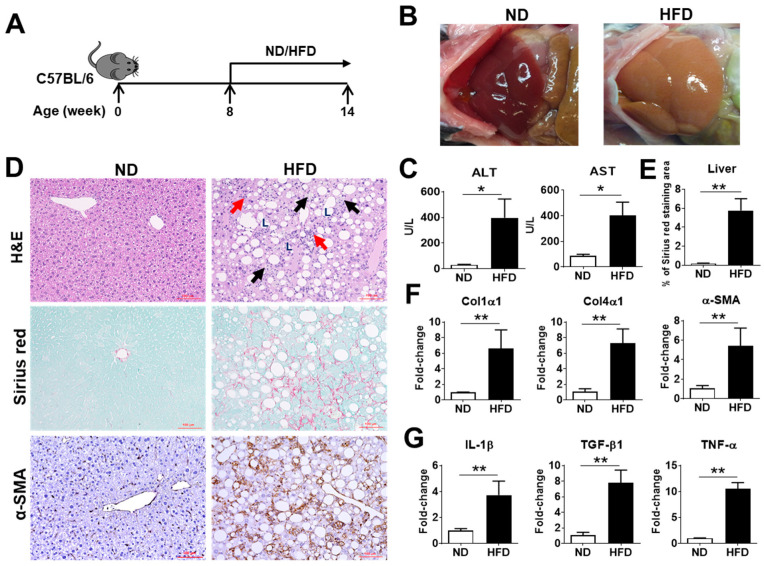
Characterization of a mouse NASH model. (**A**) An outline depicting HFD-induced mouse NASH. Eight-week-old wild-type (WT) C57BL/6 mice were fed a CDAHFD (abbreviating to HFD in the figures) for 6 weeks. Normal diet (ND) was used as a control. At week 14, each mouse was euthanized for the following studies. (**B**) Representative macroscopic images of livers in mice fed with HFD and ND. (**C**) Serum levels of ALT and AST in mice fed with HFD and ND. (**D**) Representative histological images of liver tissue in mice fed with HFD and ND. H&E staining showed lipid deposition (L), hepatocytes ballooning (black arrow), and inflammatory cell liver infiltration (red arrow); Sirius red staining showed the increased production of collagen (red staining); IHC showed the increased protein production of α-SMA (brown staining). Bar scale: 100 μm. (**E**) Semi-quantification of collagen production in the livers of two groups of mice as shown in (**D**). (**F**) mRNA expression of extracellular matrix (ECM) genes in the livers. qPCR detected the increased mRNA expression of *Col1α1*, *Col4α1*, and *α-SMA* genes in the livers of the mice fed HFD compared to ND. (**G**) mRNA expression of proinflammatory cytokines in the livers. qPCR detected the increased mRNA expression of *IL-1β*, *TGF-β1*, and *TNF-α* genes in the livers of HFD-fed mice compared to that in ND-fed mice. *n* = 5, error bars represent the mean ± SD. Statistical analysis of data was performed by Student’s *t*-test using GraphPad Prism 8 software. ** *p* < 0.01, * *p* < 0.05.

Dietary rodent models are commonly used to study the pathogenesis and therapeutic approaches of NAFLD/NASH due to the convenience, cost, and clinical relevance [28]. In this study, a CDAHFD was selected to induce mouse NASH to investigate the role of ASTN in NASH progression. Eight-week-old, wild-type C57BL/6 mice were fed with this HFD for 6 weeks (Figure 2A) to induce NASH progression. Macroscopic observation revealed that feeding HFD caused a significant size increase and yellow color change in the livers of mice (Figure 2B). Blood biochemistry examination showed a significant increase in serum levels of alanine aminotransferase (ALT) and aspartate aminotransferase (AST) in mice with NASH compared to that in ND-fed mice (Figure 2C). H&E staining revealed a noticeable increase in the infiltration of inflammatory cells, accumulation of lipid droplets, and hepatocyte ballooning (Figure 2D). Sirius red staining demonstrated that HFD treatment increased the expression of collagen, resulting in periportal and bridging fibrosis (Figure 2D). Immunohistochemistry (IHC) staining displayed a dramatically increased expression of α-smooth muscle actin (α-SMA), an activation marker of HSCs (Figure 2D,E). Real-time (qPCR) detected a significant increase in the mRNA expression of extracellular matrix (ECM) genes including *Col1α1*, *Col4α1*, and *α-SMA* (Figure 2F) and inflammatory cytokine genes including *IL-1β*, *TGF-β1*, and *TNF-α* (Figure 2G). Similar microscopic and gene level changes were observed in human fibrotic livers [29]. These results show that this HFD-induced murine NASH model shares the key features with human NASH, including steatosis, infiltration of inflammatory cells, hepatocyte ballooning injury, and liver fibrosis. 

### 2.2. ASTN Inhibits the Progression of NASH

**Figure 3:**
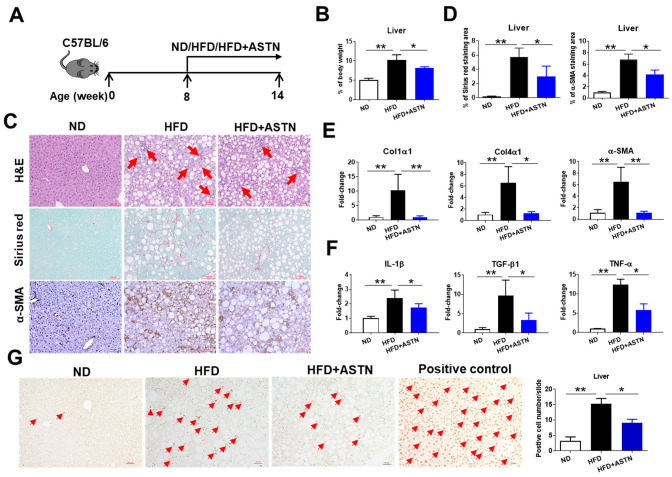
Astaxanthin treatment slows the development of HFD-induced NASH. (**A**) An outline depicting ASTN treatment design. Eight-week-old WT C57BL/6 mice were fed with a HFD for 6 weeks with or without receiving simultaneous ASTN treatment, at a dose of 80 mg/Kg of bodyweight. WT mice fed with ND were used for control. (**B**) Effect of ASTN on HFD-caused the increase in the ratios of liver-to-bodyweight. Compared to ND, HFD caused the increase in the ratio of liver-to-bodyweight which was suppressed by ASTN treatment. (**C**) Effect of ASTN on HFD-induced NASH. Compared to nontreatment, ASTN treatment led to the obvious reduction in the liver infiltration of inflammatory cells (red arrow, H&E staining) and production of the collagen (Sirius red staining) and α-SMA (IHC detection) in HFD-fed mice. Bar scale: 100 μm. (**D**) Semi-quantification of collagen production and α-SMA protein expression in the livers of three groups of mice as shown in (**C**). (**E**) ASTN reduced hepatic mRNA expression of ECM genes in HFD-fed mice. qPCR measured the reduced mRNA expression of *Col1α1*, *Col4α1*, and *α-SMA* genes in the livers of ASTN-treated mice compared to nontreatment mice. (**F**) ASTN reduced hepatic mRNA expression of proinflammatory and profibrotic cytokines. qPCR measured the reduced mRNA expression of *IL-1β*, *TGF-β1*, and *TNF-α* genes in the livers of ASTN-treated mice compared to nontreatment mice. (**G**) ASTN suppressed hepatocyte death (red arrows). Bar scale: 50 μm. Representative and accumulated results from the TUNEL assay indicated that ASTN treatment reduced dead hepatocytes in the livers of HFD-fed mice. *n* = 5, error bars represent the mean ± SD. Statistical analysis of data was performed by one-way analysis of variance (ANOVA) using GraphPad Prism 8 software. ** *p* < 0.01, * *p* < 0.05.

To test the preventive effect of ASTN on HFD-induced NASH (Figure 3A), oral administration of ASTN (80 mg/kg of mouse bodyweight) was performed simultaneously while feeding HFD. ASTN treatment reduced the HFD-induced increase in liver-to-bodyweight ratio (Figure 3B). ASTN treatment also significantly inhibited hepatic infiltration of inflammatory cells and the protein production of collagen and α-SMA in HFD-fed mice (Figure 3C,D). In addition, qPCR detected a significant reduction in hepatic mRNA expression of ECM genes including *Col1α1*, *Col4α1*, and *α-SMA* (Figure 3E) and inflammatory cytokine genes including *IL-1β*, *TGF-β1*, and *TNF-α* (Figure 3F) in HFD-fed mice once receiving ASTN treatment. Furthermore, ASTN treatment markedly inhibited HFD-induced hepatocyte death in HFD-fed mice (Figure 3G). 

### 2.3. ASTN Inhibits the Progression of Liver Fibrosis

**Figure 4:**
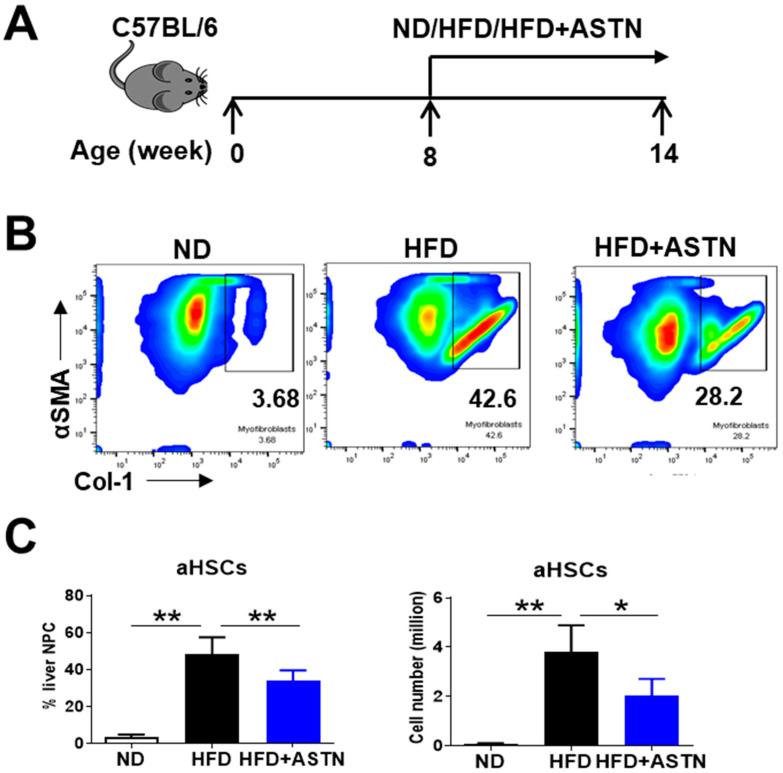
Astaxanthin treatment inhibits HFD-induced liver fibrosis. (**A**) An outline depicting ASTN treatment design. Eight-week-old WT C57BL/6 mice were fed with a HFD for 6 weeks with or without receiving simultaneous ASTN treatment. WT mice fed with ND were used for control. (**B**) Representative and (**C**) accumulated results from flow cytometric assay indicated that ASTN treatment caused the reduced frequency and cell number of activated HSCs expressing Col-1 and α-SMA in the livers of HFD-fed mice. *n* = 5, error bars represent the mean ± SD. Statistical analysis of data was performed by one-way ANOVA using GraphPad Prism 8 software. ** *p* < 0.01, * *p* < 0.05.

Activated HSCs contribute to approximately 82–96% of liver myofibroblasts in toxic and fatty liver disease models [30]. The frequency of myofibroblasts/activated HSCs (aHSCs) expressing α-SMA and Col-1 were monitored in fibrotic livers [31]. ASTN treatment significantly decreased the frequency and cell number of aHSCs (Figure 4) in mice with HFD-induced NASH. Similarly, ASTN treatment inhibited the production of collagen in CCl_4_-induced liver fibrosis (data not shown). 

### 2.4. ASTN Modulates Intrahepatic Immunity

**Figure 5:**
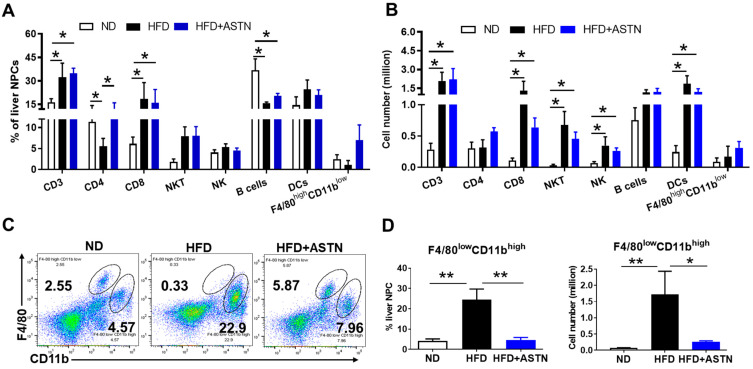
Astaxanthin treatment modulates the intrahepatic immune response. Liver non-parenchymal cells (NPCs) consisting of most CD45^+^ cells were isolated from ND, HFD, and HFD + ASTN-treated mice, and were stained with fluorochrome-conjugated antibodies. Flow cytometry was applied to analyze the components of immune cells in the livers. (**A**) The frequency of different types of immune cells in the liver. Flow cytometry was applied to define the frequency of different types of immune cells including CD3 (CD3^+^), CD4 (CD4^+^CD3^+^), CD8 (CD8^+^CD3^+^), NK (CD49b^+^CD3^‒^), NKT (NK1.1^+^CD3^+^), DCs (CD11c^+^CD11b^+^), B cells (CD3^‒^B220^+^), and liver macrophages including F4/80^high^CD11b^low^ Kupffer cells and F4/80^low^CD11b^high^ monocytes-derived macrophages. (**B**) The absolute cell number of each lymphocyte shown in (**A**). (**C**) Representative frequency of liver macrophages in NPCs. (**D**) Mean frequency (left) and absolute number (right) of F4/80^low^CD11b^high^ monocytes-derived macrophages in NPCs in three groups of mice with the indicated treatments. *n* = 5, error bars represent the mean ± SD. Statistical analysis of data was performed by one-way ANOVA using GraphPad Prism 8 software. ** *p* < 0.01, * *p* < 0.05.

Intrahepatic immunity has been demonstrated to be modulated by environmental factors (e.g., gut microbiota and diet) to influence the activation of HSCs and NAFLD/NASH progression [32]. To better understand how ASTN can orchestrate intrahepatic immunity to influence HFD-induced progression of NASH and liver fibrosis, the frequency and cell number of liver non-parenchymal cells (NPCs) in HFD-fed mice were assessed by flow cytometry. In fatty livers of NASH mice, HFD treatment caused a significant increase in both the frequency and cell number of immune cells, including CD3^+^ T cells, CD8^+^ T cells, NK1.1^+^CD3^+^ natural killer T cells (NKT), and F4/80^low^CD11b^high^ monocytes-derived macrophages (Figure 5A–D), as well as the number of CD49b^+^CD3^‒^ natural killer (NK) cells (Figure 5B). In contrast, HFD treatment resulted in a decrease in the frequency of CD4^+^ T cells and CD3^‒^B220^+^ B cells (Figure 5A), but not their cell numbers (Figure 5B). The cell number of CD11c^+^CD11b^+^ dendritic cells (DCs) (Figure 5B), but not the frequency (Figure 5A), was increased in HFD-treated mice. The frequency and cell number of F4/80^high^CD11b^low^ Kupffer cells (liver resident macrophages) were not changed (Figure 5A,B). ASTN treatment altered the effect of HFD-induced immune response. Specifically, ASTN treatment led to an increase in the frequency of CD4^+^ T cells (Figure 5A), but not their cell number (Figure 5C), and induced a decrease in frequency and cell number of F4/80^low^CD11b^high^ monocytes-derived macrophages (Figure 5C,D). 

**Figure 6:**
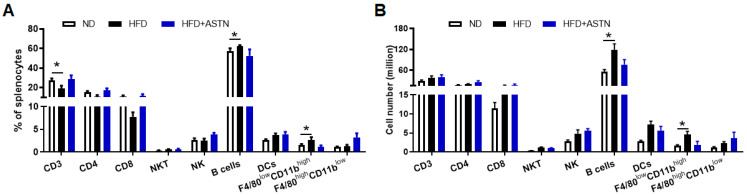
Astaxanthin treatment does not significantly modulate the systemic immune response. Spleen lymphocytes with lysis of erythrocytes were isolated from spleens of ND, HFD, and HFD + ASTN-treated mice simultaneously as liver tissues in Figure 5, stained with fluorochrome-conjugated antibodies, and analyzed via flow cytometry. (**A**) The mean frequency and (**B**) the mean absolute cell number of different types of immune cells in the spleen, including CD3 (CD3^+^), CD4 (CD4^+^CD3^+^), CD8 (CD8^+^CD3^+^), NKT (NK1.1^+^CD3^+^), NK (CD49b^+^CD3^−^), B cells (CD3^‒^B220^+^), DCs (CD11c^+^CD11b^+^), and F4/80^high^CD11b^low^ and F4/80^low^CD11b^high^ macrophages. *n* = 5, error bars represent the mean ± SD. Statistical analysis of data was performed by one-way ANOVA using GraphPad Prism 8 software. * *p* < 0.05.

To evaluate whether HFD treatment influences systemic immune response, spleen lymphocytes were isolated by lysing erythrocytes and analyzed using flow cytometry. HFD treatment significantly increased the frequency and cell number of CD3^‒^B220^+^ B cells and F4/80^low^CD11b^high^ monocytes-derived macrophages (Figure 6A,B), while it decreased the frequency of CD3^+^ T cells (Figure 6A). ASTN treatment showed a reversal effect to HFD-induced change of spleen lymphocytes, inducing a slight increase in CD3^+^ T cells and a moderate decrease in CD3^‒^B220^+^ B cells and F4/80^low^CD11b^high^ monocytes-derived macrophages in the frequency, but there was no significant change between HFD- and HFD+ASTN-treated groups (Figure 6). To summarize, ASTN treatment showed an obvious effect on modulating intrahepatic immune response via inhibiting the infiltration of monocyte-derived macrophages.

### 2.5. ASTN Inhibits Hepatic bFGF Expression and Oxidative Stress in NASH Mice

**Figure 7:**
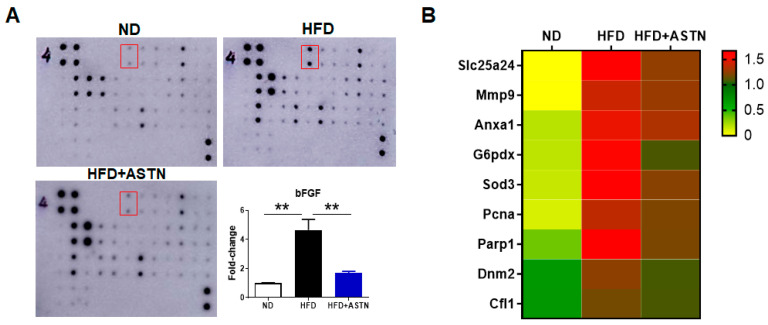
Astaxanthin treatment inhibits FGF2 in the NASH liver. (**A**) Representative images of cytokine assay. Mouse cytokine antibody array was applied to detect 34 mouse cytokines in liver tissues collected from ND-, HFD-, or HFD-ASTN-treated mice. The expression of basic fibroblast growth factor (bFGF) circled in the red box was dramatically increased in the NASH liver, which was significantly inhibited by ASTN treatment. The experiment was repeated. Error bars represent the mean ± SD. Statistical analysis of data was performed by one-way ANOVA using GraphPad Prism 8 software. ** *p* < 0.01. (**B**) Heatmap showed the expression of proteins in oxidative stress response in Biological Processes of GO: 0034599 and GO: 0006979 (online STRING software) at three indicated treatment groups.

To further investigate the molecular level of anti-NASH or liver fibrosis effect of ASTN, a cytokine array assay was performed to check cytokines that mediate NASH progression. The assay results indicated that the expression of basic fibroblast growth factor (bFGF, also known as FGF2) was dramatically increased in the NASH liver, which was significantly inhibited by ASTN treatment (Figure 7A, *p* < 0.01). In addition, some ECM-modulating proteins or cytokines in the liver were also slightly altered in HFD- and HFD-ASTN-treated groups compared to the ND-treated group. 

Oxidative stress plays an important role in NAFLD/NASH development and progression [33], associated with the change of some proteins and enzymes such as superoxide dismutase (SOD). To further explore the underlying molecular mechanism of ASTN-mediated inhibition of NASH, proteomic analysis was performed to investigate the effect of ASTN on the inhibition of oxidative stress. The results showed that HFD treatment significantly increased the oxidative stress response (Figure 7B), including proteins of mitochondrial solute carrier family 25 member 24 (Slc25a24), matrix metallopeptidase 9 (Mmp9), annexin A1 (Anxa1), glucose-6-phosphate dehydrogenase X-linked (G6pdx), superoxide dismutase 3, extracellular (Sod3), proliferating cell nuclear antigen (Pcna), poly (ADP-ribose) polymerase family, member 1(Parp1), dynamin 2 (Dnm2), and cofilin 1 (Cfl1). Conversely, ASTN treatment reduced these proteins expression in the livers of HFD-fed mice (Figure 7B). These results suggested that ASTN displays an anti-oxidative stress activity in the pathogenesis of HFD-induced NASH.

**Figure 8:**
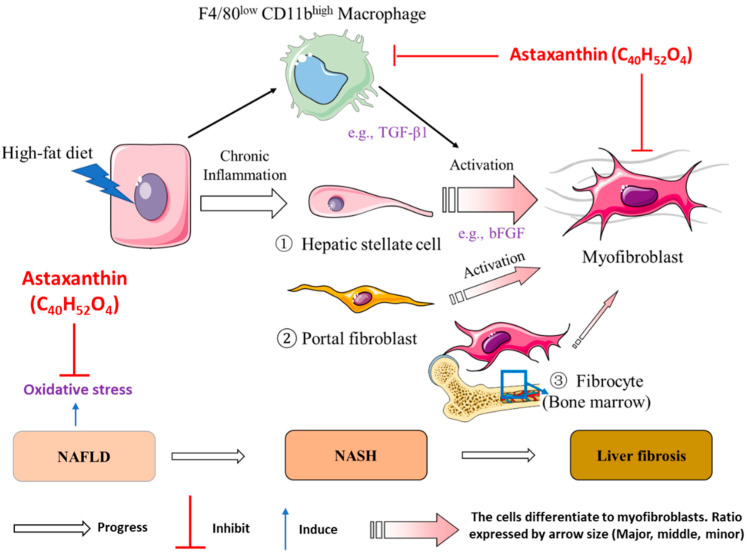
A schematic figure shows the effect of ASTN on NASH at the cellular and molecular levels. ASTN inhibits the infiltration of F4/80^low^CD11b^high^ macrophages, the expression of bFGF, and oxidative stress, resulting in retardation of liver fibrosis and NASH progression.

Overall, ASTN treatment inhibits HFD-induced NASH progression by impairing the progression of liver fibrosis, suppressing oxidative stress, and modulating intrahepatic immune response (Figure 8). Specifically, ASTN treatment can prevent the infiltration of monocyte-derived macrophages, suppress bFGF expression, and inhibit oxidative stress. 

## 3. Discussion

NASH is an increasing factor that causes the end stage of liver diseases, such as cirrhosis and HCC, posing heavy health issues and economic burden [34]. Limitation of treatment options exacerbates this burden. In this study, we explore the potential effect of a natural, antioxidant, and anti-inflammatory product ASTN on mouse NASH model [26]. The results indicated that ASTN modulates intrahepatic immunity and reduces hepatocyte death and the expression of proinflammatory genes, ECM genes, oxidative stress-associated proteins, and bFGF in the fatty liver to ameliorate NASH progression. 

ASTN has been shown to display diverse functions in metabolic disorders both in animals and humans. For example, oral administration of ASTN can improve insulin resistance and reduce blood pressure and plasma levels of triglyceride and non-esterified fatty acids in rats with auto-development of metabolic syndrome [35]. Similarly, ASTN treatment significantly reduced blood levels of glucose and total cholesterol in streptozotocin-induced diabetic rats [36]. In comparison to vitamin E, ASTN treatment showed more efficacy in reducing lipid accumulation in hepatocytes and improving insulin resistance in both genetical (ob/ob) mice and a high-cholesterol and high-fat diet-induced obese mice [37]. A recent randomized, placebo-controlled trial investigation showed that supplementation of ASTN in patients with type 2 diabetes, at a dose of 8 mg per day for 8 weeks, significantly enhanced serum levels of adiponectin and reduced serum triglyceride and very-low-density lipoprotein cholesterol concentration, blood pressure, and visceral body fat accumulation, without adverse effect compared to placebo control [38]. 

Furthermore, recent studies show that ASTN protects against liver injury, inflammation, and liver fibrosis. For example, ASTN treatment ameliorated arsenic-induced liver injury in rats via reducing the arsenic-induced increase in inflammatory cytokines NF-κB, TNF-α, and IL-1β [39]. In this study, we also have demonstrated that ASTN treatment prevents the gene expression of proinflammatory cytokines, such as *IL-1β* and *TNF-α* in the NASH liver. The anti-inflammatory and antioxidant effects of ASTN have also been illustrated in lipopolysaccharide (LPS)-activated mouse macrophage cell line RAW264.7 cells and LPS-treated mice via inhibiting nuclear factor Kappa B (NF-κB) activity [40]. The in vitro effect of ASTN on macrophages is also found in our study, which shows that ASTN can reduce the gene expression of *IL-1β*, *TGF-β1*, and *TNF-α* in LPS-activated RAW 264.7 cells (Figure S1). In addition, ASTN treatment suppressed the gene expression of chemokine CCL2 (Figure S1), which attracts the migration of monocytes or macrophages expressing CCL2 ligand CCR2 into the liver. CCL2 and CCR2 have been shown to be overexpressed in the livers of NASH patients and mice with steatohepatitis and fibrosis. In addition, pharmacologically inhibiting CCL2 expression in mice with CCl_4_ or methionine-choline-deficient (MCD) diet-induced chronic liver injury suppresses monocyte/macrophage migration into the injury liver, resulting in amelioration of hepatic steatosis and inflammation [41].

In addition to the NF-κB signaling pathway, ASTN can downregulate the c-Jun N-terminal kinase (JNK)/p-JNK signaling pathway to attenuate concanavalin A (ConA)-induced hepatitis and reduced serum liver enzymes in mice [42]. Additionally, also, ASTN treatment is able to attenuate free fatty acid-induced lipid accumulation and cell apoptosis in human liver cell line L02 cells, and reduce liver inflammation, hepatocyte damage, and mitochondrial dysfunction in HFD-fed mice, via up-regulating fibroblast growth factor 21 (FGF21) and Peroxisome proliferator-activated receptor-gamma coactivator-1 alpha (PGC-1α) expression [43]. However, the role of ASTN in modulating hepatic and systemic immune response in NASH model remains to be further explored.

Intrahepatic immunity has been shown to play a critical role in the progression of NASH and liver fibrosis. Among the intrahepatic immune cells, macrophages including Kupffer cells and other sources of macrophages predominated by monocytes-derived macrophages play critical roles in NASH progression [44]. F4/80^high^CD11b^low^ macrophages and F4/80^low^CD11b^high^ macrophages are defined as Kupffer cells and monocyte-derived macrophages, respectively) [45]. Here, we showed that ASTN functions on intrahepatic immune cells to inhibit NASH progression and liver fibrosis, specifically by suppressing F4/80^low^CD11b^high^ monocytes-derived macrophages (Figure 5). A study also showed that ASTN treatment increased M2 macrophages with markers such as CD206 and IL-10, while it decreased M1 macrophages with markers such as CD11c and CCR2 in a high-fat, cholesterol, and cholate diet-induced mouse NASH model [37]. Another very recent study also showed that in combination with β-Cryptoxanthin, a xanthophyll carotenoid, ASTN inhibited inflammation and promoted M2 macrophage polarization, anti-inflammation phenotype [46]. 

Our data also showed that T cells are also implicated in NASH progression, which is consistent with recent findings. For example, liver CXCR6^+^CD8^+^ T cells are susceptible to metabolite stimuli, and their accumulation is positively associated with mouse and human NASH development [47]. In addition, the increase in hepatic PD1^+^CXCR6^+^CD8^+^ T cells during NASH-HCC progression impairs anti-tumor immunotherapy against HCC [48]. However, we did not find the ASTN treatment had an obvious impact on whole hepatic CD3^+^, CD4^+^, and CD8^+^ T cell frequency and cell number in this model. The effect of ASTN treatment on each subtype of liver T cells is considered in the future. Furthermore, we also found that the frequency of hepatic B cells reduced due to an increase in infiltration of other immune cells, whereas their cell number slightly increased (Figure 5A,B). In contrast, both the frequency and cell number of spleen B cells increased in HFD-fed mice compared to ND-fed mice, but ASTN did not show a significant effect to reverse this change (Figure 6A,B). A recent study showed that gut-microbiota-mediated activation of B cells contributed to NASH development [49], which is associated with an increase in inflammation and oxidative stress [50]. However, since there was no significant effect of ASTN on modulating T and B cells, this study did not focus on these populations. 

Activated HSCs mainly contribute to myofibroblasts in the liver, resulting in fibrogenesis. This study showed that ASTN treatment dramatically ameliorates HFD-induced frequency and cell number of α-SMA^+^Col-1^+^ myofibroblasts or activated HSCs during NASH progression. It also showed that FGF2 (or bFGF) is overexpressed in the NASH liver, which has been shown to be critical for the development of liver fibrosis [51,52] and HCC [53,54,55]. In addition, ASTN inhibits the gene expression of *TGF-β1*, a predominant profibrotic gene for HSC activation [56]. The anti-fibrotic effect of ASTN is consistent with its effect on fibrotic livers in mice treated CCl_4_ or BDL [23], and in rats with arsenic-induced liver injury [39]. 

Enhancing the therapeutic effect or bioactivity of ASTN has been investigated in animal studies. For example, Gao et al. reported that ASTN and ASTN n-octanoic acid diester (AOD), a type of medium-chain fatty acids containing ASTN, modulated gut microbiota composition in high-fat and high-sucrose diet-fed mice [57]. Specifically, AOD treatment significantly increased the abundance of *Bacteroides* and *Coprococcus* in mice compared to that in ASTN-treated mice, showing a better effect in improving insulin resistance, systematic and intestinal inflammation, and intestinal integrity. Nanoparticles, applying to deliver ASTN and to improve its absorbance, have been studied in different models, such as via ASTN-polyethylene glycol grafted chitosan [58] and via self-nano-emulsifying drug delivery systems [59]. Together, these results suggest that ASTN displays multiple functions on inhibiting NASH progression via modulating intrahepatic immunity, which can be improved via modulating the delivery format. 

## 4. Materials and Methods

### 4.1. Animals, Diets, and Murine NASH Model

Male C57BL/6 mice were bought from Jackson Laboratory (Bar Harbor, ME, USA). A choline-deficient, L-amino acid-defined, high-fat diet (CDAHFD, A06071302, Research Diets Inc., New Brunswick, NJ, USA) was fed to induce mouse NASH with rapid liver fibrosis development. CDAHFD (short for HFD) consists of 60 kcal% fat and 0.1% methionine by weight [26]. Eight-week-old C57BL/6 mice were randomly grouped and fed with either ND or HFD as the indicated times, five mice per group. For testing the effect of ASTN on NASH progression, oral administration of ASTN at a dose of 80 mg/Kg mouse bodyweight was performed triplicates each week for 6 weeks while feeding HFD. This dose of ASTN showed a better anti-fibrotic effect in CCl_4_-treated mice [23]. All experiments involving animals were approved by the Animal Care and Use Committee of the University of Missouri (protocol code: 9475, and approved date: 11/2018–11/2021). All mice received humane care according to the criteria outlined in the “Guide for the Care and Use of Laboratory Animals”.

### 4.2. Cell Lines, Bacterial Strains, and Medium

Mouse macrophage cell line RAW264.7 (TIB-71, ATCC, Manassas, VA, USA) was cultured in Dulbecco’s Modified Eagle’s Medium (DMEM), respectively. The full culture medium was supplemented with 10% of fetal bovine serum (FBS), 100 U/mL penicillin, and 100 μg/mL streptomycin at 37 °C in a humidified atmosphere with 5% of CO_2_. All the DMEM, FBS, and penicillin/streptomycin antibiotic solutions were purchased from Thermo Fisher Scientific (Waltham, MA, USA).

### 4.3. Blood Biochemistry Test

The enzymes AST and ALT were used to assess liver function and lipid metabolism. Blood biochemical profile analysis was carried out using the Olympus 400AUe Chemistry Analyzer (Olympus Corporation, PA, USA) in the Veterinary Medical Diagnostic Laboratory at the University of Missouri.

### 4.4. Total RNA Extraction and Real-Time PCR (qPCR)

Total RNA was extracted with RNeasy@ Micro kit (Qiagen, Germantown, MD, USA) according to the manufacturer’s instructions. Reverse transcription of total RNA to cDNA was conducted with a High-Capacity cDNA Reverse Transcription kit (Applied Biosystems, Foster, CA, USA). qPCR was performed with QuantStudio 3 Detection System (Thermo Fisher, Waltham, MA, USA) in a 20 μL reaction mixture containing SYBR Green PCR Master Mix (Thermo Fisher, Waltham, MA, USA). Reactions were run in triplicate. Expression of different genes was normalized to the geometric mean of housekeeping gene 18S RNA for controlling the variability of expression levels. The data were analyzed using the 2^−ΔΔCT^ method. All primers were synthesized by Integrated DNA Technologies, Inc. (Skokie, IL, USA) and their sequences were listed in Table S1.

### 4.5. Isolation of Liver-Infiltrating Leukocytes and Spleen Lymphocytes

The isolation of liver-infiltrating leukocytes has been described in our previous publication [60]. Briefly, liver perfusion was first performed in mice under anesthesia via the portal vein with 0.05% collagenase (Gibco, Gaithersburg, MD, USA) in Ca^2+^-free PBS at a pump speed of 4 mL/min. The harvested live tissues were cut to small pieces and incubated in 0.04% collagenase in GBSS (Sigma, St. Louis, MO, USA) at room temperature with continuous shaking at a speed of 240 rpm. Twenty minutes later, the suspended samples were filtered through 250 µm mesh, then centrifuged at 350× *g* for 10 min at room temperature to harvest cell pellets. The cell pellets were suspended and washed with GBSS (Sigma, St. Louis, MO, USA). After centrifugation, the harvested cells were suspended in 15 mL of GBSS and mixed with 18.45 mL of 30% Nycodenz solution (Accurate Chemical & Scientific Inc., Westbury, NY, USA). The cell suspension underwent gradient centrifugation at 1400× *g* for 20 min at room temperature with no brake. The enriched leukocytes in the top layer were harvested and washed with PBS, then suspended in flow cytometry buffer for the following experiments. The frequency of each type is the percentage of this cell in the total of liver NPCs.

The freshly isolated spleen was meshed with the plunger end of a 5 mL syringe, then cell suspension was passed through a 40 μm cell strainer. Red blood cells were removed with eBioscience™ 1× RBC Lysis Buffer (ThermoFisher Scientific, Waltham, MA, USA). Mixed spleen cells were washed with PBS, then suspended flow cytometry buffer for flow cytometry analysis. The frequency of each type of lymphocyte is the percentage of this cell in the total of mixed spleen cells.

### 4.6. Flow Cytometry

Ex vivo staining of lymphocytes with fluorochrome-labeled antibodies was performed on single-cell suspensions as described [60]. For intracellular staining, the cells were fixed and permeabilized with buffer (ThermoFisher Scientific, Waltham, MA, USA), and stained with fluorochrome-conjugated antibodies. Stained cells were analyzed with a FACScan Flow Cytometer (BD Biosciences, San Jose, CA, USA). Data were analyzed using FlowJo software version 10.7.1 (Tree Star, Ashland, OR, USA). The sources of all antibodies used in this experiment were listed in Table S2.

### 4.7. H&E Staining

Formalin-fixed paraffin-embedded (FFPE) liver blocks were used to make 4 μm tissue sections. The staining with hematoxylin and eosin (H&E) was performed in the Veterinary Medical Diagnostic Laboratory at the University of Missouri.

### 4.8. Liver Collagen Staining

A Sirius red kit (Chondrex, Redmond, WA, USA) was used to stain collagen in the liver tissue section for liver fibrosis identification according to the manufacture’s instruction. Areas of positively stained sites were measured in 5 randomly selected fields of each slide with ImageJ software (National Institutes of Health, Bethesda, MD, USA).

### 4.9. Immunohistochemical Staining (IHC)

Formalin-fixed paraffin-embedded (FFPE) blocks were used to make 4 μm tissue sections. To conduct IHC, tissue sections were de-paraffinized with xylene, rehydrated with various grades of ethanol (100%, 95%, 80%, and 70%), antigen unmasked with solution (Vector Laboratories Inc., Burlingame, CA, USA), permeabilized with 0.2% Triton X-100, blocked with serum, then incubated with BLOXALL reagent (Vector Laboratories Inc., Burlingame, CA, USA) to quench endogenous peroxidase. Subsequently, the sections were incubated in succession with primary antibodies at optimized concentration, secondary antibody, and DAB substrate to develop color. The number of positive cells was counted in 5 randomly selected fields in each slide with ImageJ software.

### 4.10. TUNEL Assay

TUNEL (terminal deoxynucleotidyl transferase dUTP nick-end labeling) staining was performed using a Click-iT™ TUNEL Colorimetric IHC Detection Kit (C10625, Invitrogen, Carlsbad, CA, USA) according to the manufacturers’ instructions.

### 4.11. Cytokine Array Assay

Mouse Cytokine Antibody Array C4 Kit (RayBiotech Inc., Peachtree Corners, GA, USA) was applied to detect 34 mouse cytokines in liver tissues collected from ND-, HFD-, or HFD-ASTN-treated mice. The procedure was performed according to the manufacture’s protocol. Images of staining membrane were captured using Amersham Imager 600 (GE Healthcare, Marlborough, MA, USA), and the images were analyzed by ImageJ software.

### 4.12. Proteomic Analysis

Liver biopsies isolated from ND-, HFD-, or HFD-ASTN-treated mice were immediately frozen in liquid nitrogen and sent to Gehrke Proteomics Center for proteomic analysis (University of Missouri, Columbia, MO, USA). Significantly changed proteins were further analyzed for searching proteins involved in the biological process of oxidative stress response using online software STRING (https://string-db.org/ (accessed on 30 September 2021)).

### 4.13. Statistical Analysis

Statistical significance between groups was determined using one-way ANOVA (≥3 groups) or Student’s *t*-test followed by the Mann–Whitney test (two groups) using GraphPad Prism 8.3.0 software (GraphPad Software, La Jolla, CA, USA). Data are represented as the mean ± SD. A *p*-value of less than 0.05 was considered a significant difference.

## 5. Conclusions

In conclusion, we have illustrated more functional effects of ASTN on a diet-induced mouse NASH model. ASTN treatment can modulate intrahepatic immunity, particularly suppressing the infiltration of monocyte-derived macrophages, which may be associated with its effect on suppressing pro-inflammatory and oxidative stress response in the liver and CCL2 expression in macrophages. In addition, ASTN treatment significantly reduces the frequency and cell number of activated HSCs or myofibroblast in the NASH liver, resulting in a reduction in liver fibrosis. These effects are associated with that ASTN ameliorates hepatic expression of proinflammation (e.g., IL-1) and profibrotic genes (e.g., TGF-β1) and bFGF. In addition, current research studies aim to improve the efficacy of ASTN via administrating strategy. These findings suggest that ASTN is a potential therapeutic agent for NASH and liver fibrosis.

## Data Availability

All the data supporting reported results can be found in this manuscript.

## Supplementary Materials

The Supplementary Materials are available online at https://www.mdpi.com/article/10.3390/ijms222011037/s1.
