# Supplementary material for: Astaxanthin Prevents Diet-Induced NASH Progression by Shaping Intrahepatic Immunity
Source: Int J Mol Sci. 2021 Oct 13;22(20):11037. doi: 10.3390/ijms222011037 (PMC8541356; doi:10.3390/ijms222011037)
Supplement: Supplementary file 1 [file ijms-22-11037-s001.zip › ijms-1395531-supplementary.pdf]

International Journal of *Molecular Sciences*

MDPI

Supplementary Information

Astaxanthin Prevents Diet-Induced NASH Progression by Shaping Intrahepatic Immunity

Ming Yang [1], Eric T. Kimchi [1,2], Kevin F. Staveley-O'Carroll[1,2,*], and Guangfu Li [1,2,3,*]

[1] Department of Surgery, University of Missouri, Columbia, MO 65212, USA; yangmin@health.missouri.edu
[2] Harry S. Truman Memorial VA Hospital, Columbia, MO 65201, USA; kimchie@health.missouri.edu
[3] Department of Molecular Microbiology and Immunology, University of Missouri, Columbia, MO 65212, USA;
* Correspondence: K.S., ocarrollk@health.missouri.edu; G.L., liguan@health.missouri.edu.

Supplementary materials

Table S1 Primers for real-time PCR.

Genes	Forward (5′-3′)	Reverse (5′-3′)
α-SMA	GGCTCTGGGCTCTGTAAGG	CTCTTGCTCTGGGCTTCATC
Col1α1	CCAAGGGTAACAGCGGTGAA	CCTCGTTTTCCTTCTTCTCCG
Col4α1	TTAAAGGACTCCAGGGACCAC	CCCACTGAGCCTGTCACAC
IL-1β	TCTGAAGCAGCTATGGCAAC	ATGAGTTGGGGACTCTCTGG
TGF-β1	GGTTCATGTCATGGATGGTGC	TGACGTCACTGGAGTTGTACGG
TNF-α	ACGGCATGGATCTCAAAGAC	GTGGGTGAGGAGCACGTAGT
CCL2	CCCCAAGAAGGAATGGGTCC	GTGCTGAAGACCTTAGGGCA
CCL5	TGCCAACCCAGAGAAGAAGTG	TAGGGGATTACTGAGTGGCATC

Table S2 Antibodies for flow cytometry and immunohistochemistry.

REAGENT or RESOURCE	SOURCE	IDENTIFIER
APC anti-mouse/human CD45R/B220 Antibody (FACS)	BioLegend	CAT# 103212
FITC anti-mouse CD3 Antibody (FACS)	BioLegend	CAT# 100204
PE anti-mouse CD4 Antibody (FACS)	BioLegend	CAT# 116006
BV605 anti-mouse CD8a Antibody (FACS)	BioLegend	CAT# 100744
BV605 anti-mouse/human CD11b Antibody (FACS)	BioLegend	CAT# 101257
FITC anti-mouse CD11c Antibody (FACS)	BioLegend	CAT# 117306
BV421 anti-mouse CD45 Antibody (FACS)	BioLegend	CAT# 103133
FITC anti-mouse CD49b (pan-NK cells) Antibody (FACS)	BioLegend	CAT# 108906
APC anti-mouse F4/80 Antibody	BioLegend	CAT# 123116
FITC anti-mouse NK-1.1 Antibody	BioLegend	CAT# 108706
7-AAD Viability Staining Solution	BioLegend	CAT# 420404
Alexa Fluor® 647 Mouse anti-GFAP	BD Biosciences	CAT# 560298
Fixable Viability Dye eFluor™ 780	Invitrogen	CAT# 65-0865-14
Collagen I alpha 1 Antibody [FITC]	Novus Biologicals	CAT# NBP1-77458F
alpha-Smooth Muscle Actin Antibody (1A4/asm-1) [PE]	Novus Biologicals	CAT# NBP2-34522PE
Anti-alpha smooth muscle Actin antibody [E184]	Abcam	CAT# ab32575

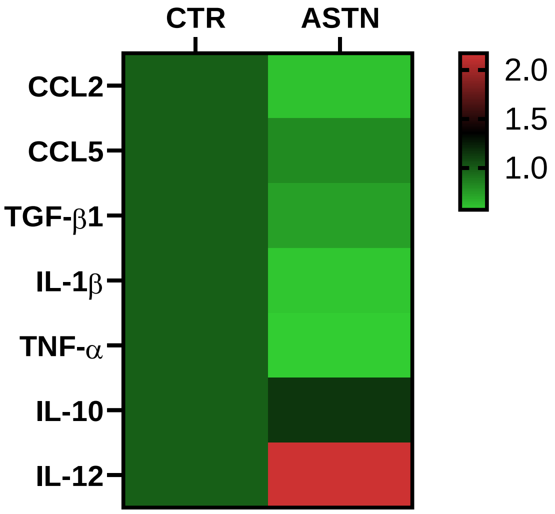

Figure S1. Astaxanthin treatment inhibits LPS-induced activation of RAW264.7 cells. RAW264.7 cells were stimulated with 100 ng/ml of lipopolysaccharide (LPS) and co-treated with ASTN or vehicle control (CTR). qPCR measured ASTN treatment decreased the mRNA expression of pro-inflammatory cytokines CCL2, CCL5, TGF-β1, IL-1β, and TNF-α, while increased the mRNA expression of anti-inflammatory cytokines IL-10 and IL-12 in RAW264.7 cells compared to the control group. n=3, error bars represent the mean ± SD. Statistical analysis of data was performed by Student's *t*-test using GraphPad Prism 8 software. $^{**}p < 0.01$, $^{*}p < 0.05$.
